# Case Report: Staged surgical repair and negative pressure wound therapy in congenital omphalocele

**DOI:** 10.3389/fped.2026.1771958

**Published:** 2026-03-18

**Authors:** Francesco Misticoni, Valentina Chiavaroli, Chiara Cauzzo, Davide Scarponcini Fornaro, Maria Enrica Miscia, Mario Fusillo, Gabriele Lisi, Francesco Chiarelli, Susanna Di Valerio

**Affiliations:** 1 Department of Pediatrics, “G. d'Annunzio” University of Chieti and Pescara, Chieti, Italy; 2 Neonatal Intensive Care Unit, Santo Spirito Hospital, Pescara, Italy; 3 Pediatric Surgery Unit, Santo Spirito Hospital, Pescara, Italy; 4 Department of Medicine and Aging Science, “G. d'Annunzio” University of Chieti and Pescara, Chieti, Italy

**Keywords:** congenital disorders, negative pressure wound therapy, newborn, omphalocele, surgical procedures

## Abstract

**Background:**

Omphalocele is a congenital abdominal wall defect that causes the protrusion of the abdominal organs at the base of the umbilical cord, which is covered by a membranous sac composed of the peritoneum and amnion. We report the case of a newborn with a giant omphalocele containing the liver and bowel loops who underwent staged surgical closure followed by negative pressure wound therapy (NPWT) for the management of skin wound dehiscence.

**Case presentation:**

A male Caucasian infant was born at 35 weeks by elective cesarean section due to a prenatal diagnosis of a giant omphalocele. The clinical examination at birth confirmed a giant omphalocele with extensive liver and small bowel content. Cardio-respiratory and neurological examinations were unremarkable. The omphalocele was initially wrapped with hydrocolloid dressings. After bowel content reduction and epithelialization of the membrane, a three-stage surgery was scheduled as follows: first, a silo bag was fashioned (on day 22); then, after complete liver reduction, the wide muscle-fascial defect was temporarily covered by a porcine dermal implant to close the gap (on day 32); finally, the abdominal wall was fully closed without prosthetic material using the component separation method (on day 68). NPWT was performed in two stages for a total of 29 days. The first period, applied between the second and third interventions, began on day 37 and continued for 16 days. Due to partial dehiscence of the surgical wound, NPWT was restarted on day 75 for 13 days, with increasing pressure (from −20 to −40 mmHg). Progressive improvement of the wound was obtained. After NPWT removal, an antimicrobial hydro-balanced dressing was placed on the wound.

**Conclusions:**

This case underlines the lack of a standardized therapeutic approach for complex abdominal wall defects. It also highlights the efficacy of NPWT in complicated surgical wounds in infancy, given the reduction in dressing frequency and related stress. Furthermore, NPWT guarantees a significant reduction in the number of days required for the resolution of the surgical wound.

## Introduction

Omphalocele is a congenital abdominal wall malformation consisting of the protrusion of abdominal organs at the base of the umbilical cord covered by a membranous sac composed of the peritoneum and amnion. Its estimated global incidence is 1 in 10,000 live births (1, 2). An omphalocele can be classified primarily based on the following:

- location: epigastric, umbilical (central), or hypogastric; and
- diameter (giant if the defect is > 5 cm and/or liver herniation) (3–7).

It often comprises bowel loops; however, the liver, spleen, and gonads can be found in the sac.

The pathogenesis appears to be related to an incorrect folding of the ventral abdominal wall during embryogenesis (8, 9).

Omphalocele can present as an isolated defect or in association with syndromes, such as trisomy (i.e., 13, 18, and 21), Beckwith–Wiedemann syndrome, Pentalogy of Cantrell, omphalocele-exstrophy-imperforate anus-spinal syndrome, other congenital defects (e.g., congenital heart disorders), diaphragmatic hernias, and bladder and cloacal exstrophy (10, 11).

Due to its complexity, a multidisciplinary approach is recommended for the management of omphaloceles, especially in giant omphaloceles. Primary closure can be attempted in small defects mainly with bowel herniation, while staged correction or non-operative management is suggested for giant omphaloceles.

Recently, the use of negative pressure wound therapy (NPWT) has been reported as helpful to manage the most complicated cases with associated wound dehiscence (12).

Herein, we report the case of a male newborn with a giant omphalocele who underwent staged surgical treatment, complicated by a dehiscent surgical wound that was successfully managed using NPWT.

## Case description

A male Caucasian infant was delivered at 35 weeks of gestation by elective cesarean section due to a prenatal diagnosis of omphalocele on fetal ultrasound performed at 13 weeks of gestation. The decision to perform a near-term cesarean section was made to prevent spontaneous delivery, further intestinal damage, and the possible development of intrauterine growth restriction. The defect contained intestinal loops and the liver. At delivery, the infant’s Apgar score was 7 at 1 min and 9 at 5 min of life. Birth anthropometry was as follows: weight of 2,139 g (14th centile), length of 43 cm (fifth centile), and head circumference of 32 cm (28th centile). The boy was the first child of unrelated healthy parents. The family history was negative for congenital malformations and disorders. The mother’s pregnancy was characterized by gestational diabetes, treated by dietary restriction, and by hypothyroidism, treated by thyroxine.

**Figure 1:**
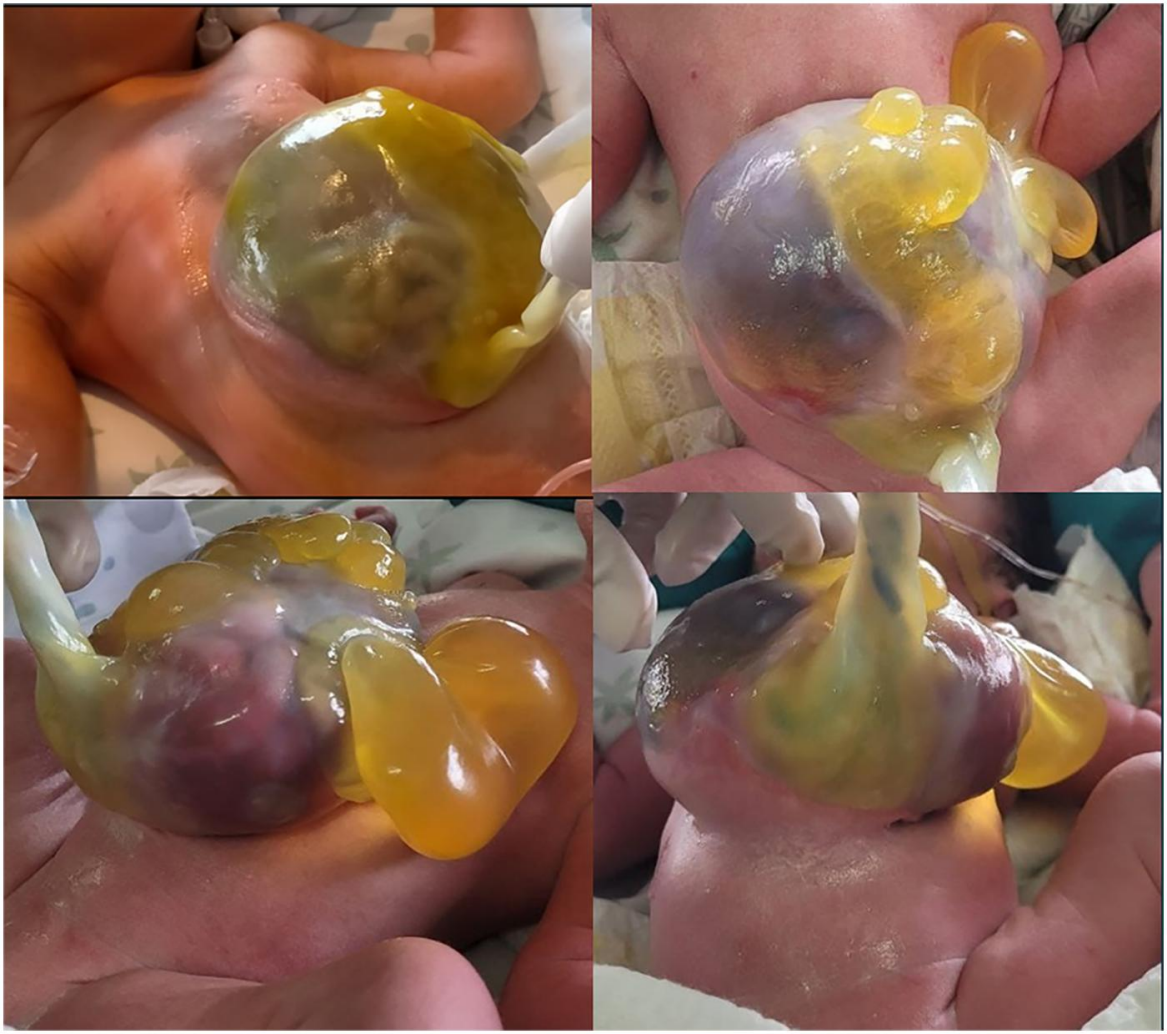
A giant neonatal omphalocele with extensive liver and small bowel herniation.

The full clinical examination at birth confirmed the diagnosis of a giant omphalocele comprising the liver and small bowel herniation (Figure 1). No other abnormalities were noted. The cardiological, abdominal, and neurological examinations were unremarkable. The newborn's general appearance and clinical conditions were otherwise normal.

### Admission to the neonatal intensive care unit

Immediately after birth, the first surgical assessment was performed. The omphalocele was wrapped with two adhesive hydrocolloid dressings (20 cm × 20 cm) to maintain the moisture balance, minimize water and heat loss, create a barrier to limit microbial contamination, and provide mechanical protection. A nasal gastric tube was placed, and rectal stimulation was performed with meconium passage. Parenteral nutrition was initiated via a peripherally inserted central catheter. Due to the occurrence of severe respiratory distress, with increased respiratory effort (i.e., polypnea), the patient was intubated, mechanical ventilatory support was started, and a dose of surfactant was administered.

### In-depth analysis

Baseline laboratory investigations, including a complete blood count, serum electrolytes, renal function tests, and liver function tests, were normal. An abdominal ultrasound revealed normal abdominal organs in shape and volume. No abnormality was found either on an ECG or echocardiography. The brain ultrasound was normal. Otoacoustic emissions were normal, as were the fundus oculi. The karyotype test was negative for a rearrangement of chromosomes 13, 18, and 21; furthermore, microdeletions and microduplications related to Beckwith–Wideman syndrome and Silver–Russell syndrome were excluded.

### Surgical management

**Figure 2:**
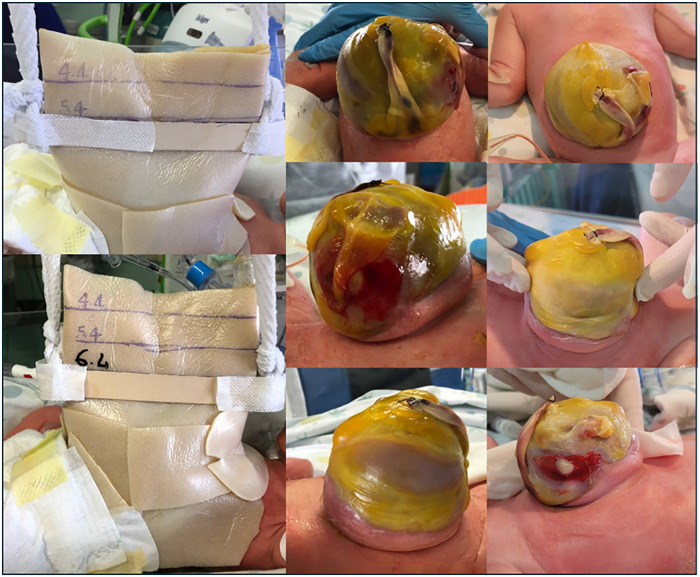
Initial progressive compression of the defect with a tongue depressor, with limited liver reduction efficacy and progressive epithelization of the outer jelly layer. The sequential images demonstrate the compression exerted by the tongue depressor and the gradual epithelialization process of the outer jelly layer occurring over the days after birth.

**Figure 3:**
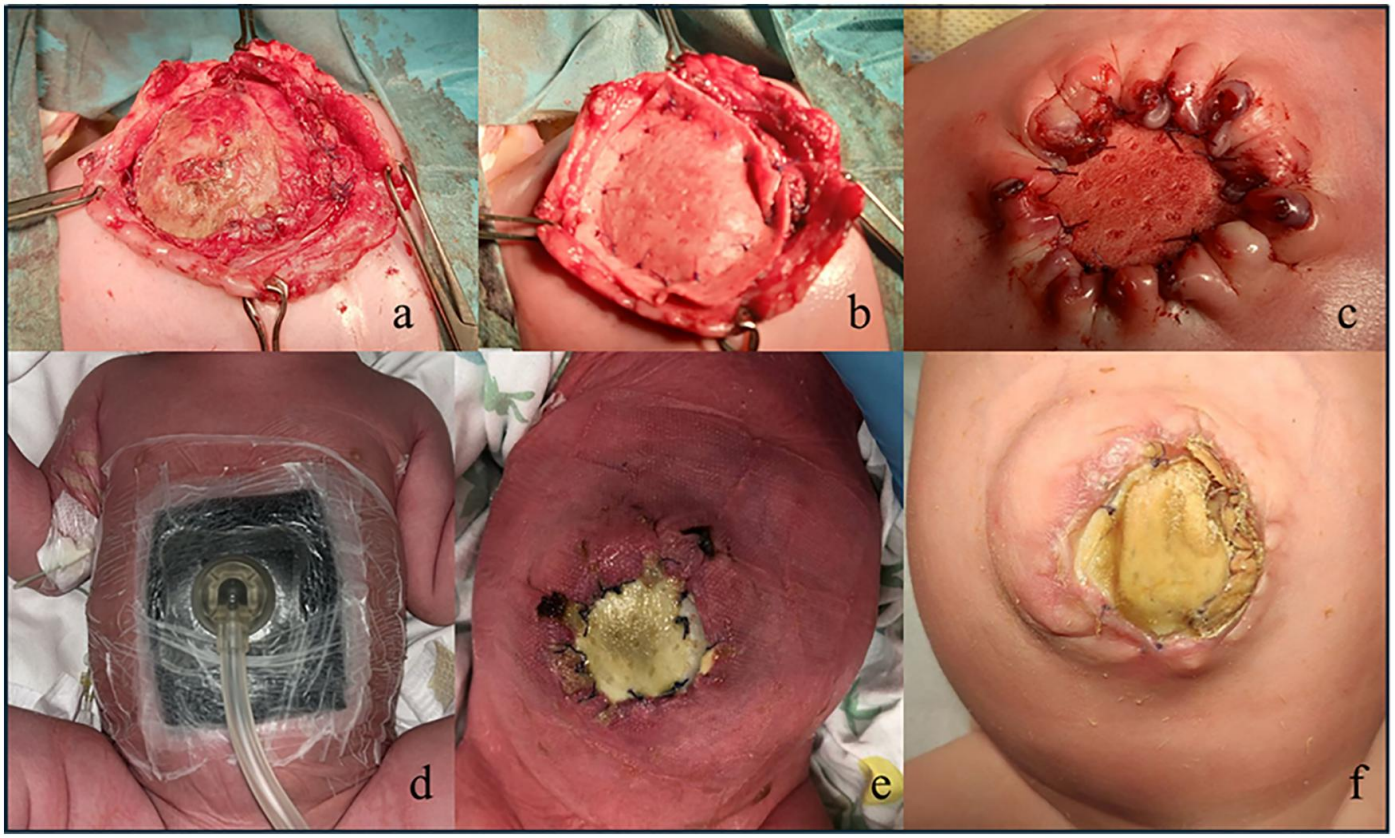
On day 32, liver reduction and abdominal defect closure were performed, with temporary positioning of a collagen-based biological implant, namely a porcine patch (Permacol, Covidien, USA), to protect the abdominal content and cover the wound in the muscle and fascia **(a–c)**. A negative pressure wound therapy device (Suprasorb® CNP P3, Lohmann & Rauscher, Germany and Austria) was applied on day 37 and continued for 16 days **(d)**, with healing progression **(e,f)**. The negative pressure wound therapy dressing consists of multiple layers, including a drainage foil (i.e., a perforated contact layer, Suprasorb® CNP) that provides uniform suction, facilitates the removal of fluids, and protects the underlying tissue.

**Figure 4:**
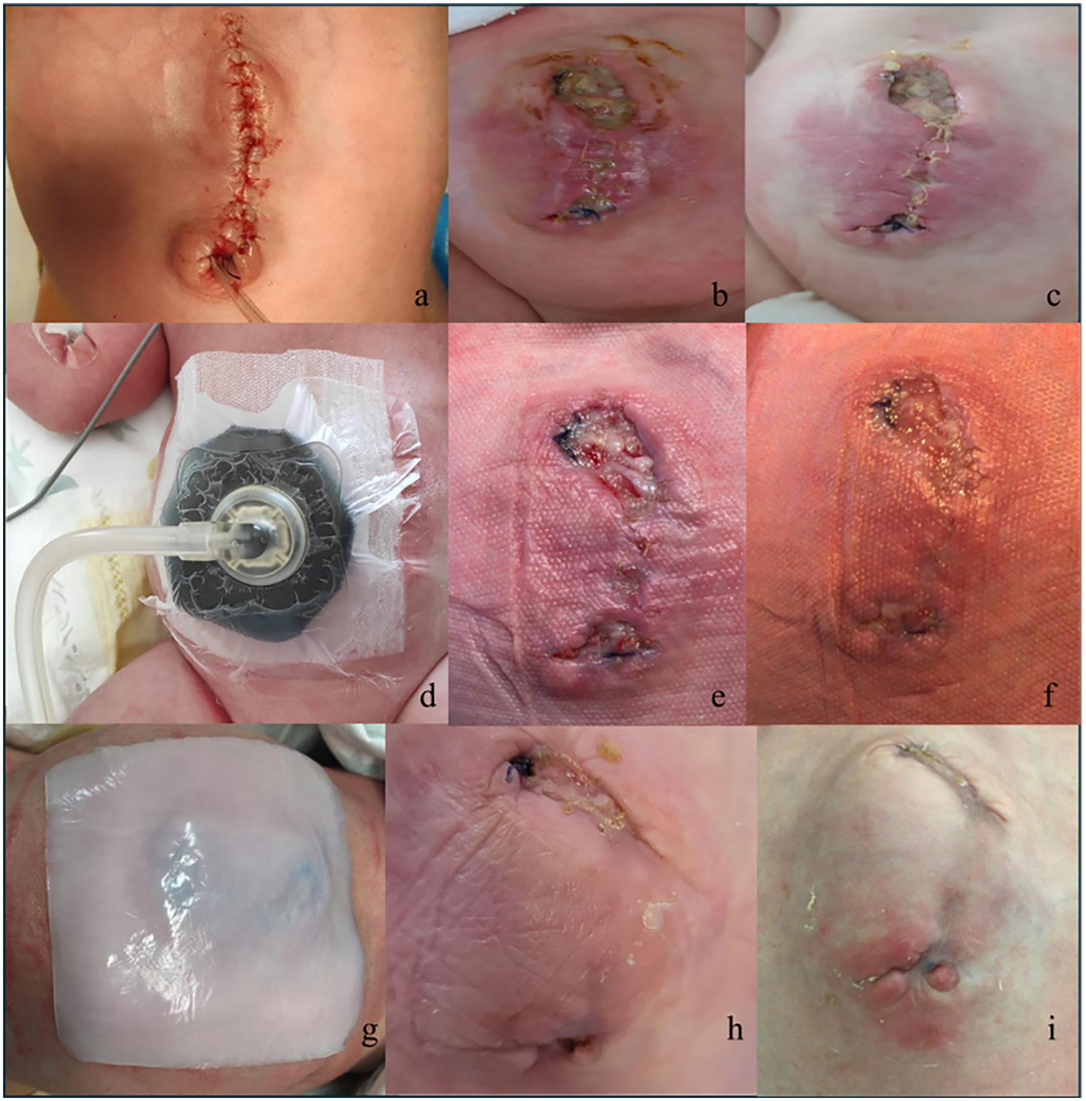
The abdominal wound was fully closed using the component separation method and umbilicoplasty was performed **(a).** Progressive cutaneous wound dehiscence was observed in the postoperative period **(b,c)**. On day 75, negative pressure wound therapy was reinstituted due to partial surgical wound dehiscence and continued for 13 days **(d)** to facilitate wound healing **(e,f)**. After removal of the negative pressure wound therapy device, a cellulose-based, moisture-balancing antimicrobial dressing (Suprasorb® X + PHMB, Lohmann & Rauscher, Germany and Austria) was placed on the wound **(g)**, with healing progression **(h,i)**.

Initial progressive compression of the defect with a tongue depressor was performed with limited liver reduction efficacy and progressive epithelization of the outer jelly layer (Figure 2). Therefore, a three-stage surgery was scheduled as follows: first, on day 22 of life, an attempt was made to close the abdominal wall. However, after the removal of the outer and jelly layers, due to the defect being giant with hepatic content, closure could not be achieved. Thus, a polypropylene surgical mesh was placed on the wound and a temporary silo bag was constructed. On day 32, the patient was taken to theater, the liver was entirely reduced, and the abdominal defect was closed with the temporary positioning of a collagen-based biological implant to protect the abdominal content and cover the wide wound in the muscle and fascia (Figures 3a–c). On day 68, after removing the porcine patch, the abdominal wall was fully closed without prosthetic material using the component separation method, and umbilicoplasty was performed (Figure 4a). The patient was extubated on day 73 of life. In the third postoperative period, progressive cutaneous wound dehiscence was observed (Figures 4b,c).

### NPWT management

NPWT was performed in two distinct stages for a cumulative duration of 29 days. A NPWT device (Suprasorb® CNP P3, Lohmann & Rauscher, Germany and Austria) was initially applied between the second and third surgical interventions (Figure 3d), commencing on day 37 and continuing for 16 days, with progressive healing (Figures 3e,f).

The NPWT involved the application of a non-adherent contact layer and foam over the sac, sealing it with adhesive film to create a vacuum. A suction pad was connected to a negative pressure unit, with continuous monitoring of the seal and organ viability.

Subsequently, on day 75, NPWT was reinstituted with the same dressing configuration for an additional 13 days to facilitate healing following the observation of partial surgical wound dehiscence (Figures 4d–f). Increasing pressure was used (from −20 to −40 mmHg), resulting in progressive improvement of the wound. After NPWT removal, a cellulose-based, moisture-balancing antimicrobial dressing (Suprasorb® X + PHMB, Lohmann & Rauscher, Germany and Austria) was placed on the wound (Figure 4g) to preserve moisture equilibrium and establish a protective barrier against microbial infiltration, with healing progression (Figures 4h,i). The neonate showed a good tolerance to NPWT.

Enteral nutrition was started on day 32, with gradual tolerance. Full enteral feeding was achieved at 80 days of life.

### Infective development

At birth, a broad-spectrum antibiotic therapy with ampicillin/sulbactam and gentamycin was started, until day 10 and day 5 of life, respectively. At birth and on day 28, blood cultures were performed, which were negative for pathogens.

On day 10, due to an increase in C-reactive protein (CRP) level, ceftazidime was started, with a resulting reduction in CRP.

On day 28, the patient presented with a fever, poor clinical condition, and an increase in CRP level; thus, antifungal therapy (amphotericin B) was started (until day 46), and the ongoing antibiotic therapy was changed to imipenem and teicoplanin, with the former maintained until day 49.

An amniotic membrane culture, a surgical device culture, and a surgical wound swab were performed prior to the surgeries (days 22, 30, and 58) and were positive for *Aspergillus flavus* (the former) and for *Staphylococcus haemolyticus* (the latter two). Therefore, the changes in the antifungal and antibiotic therapies were culture-guided. On day 69, a second cycle of ceftazidime was started and maintained until day 73, on which it was substituted with imipenem, which was maintained until day 80. Teicoplanin was stopped on day 89. CRP was negative at discharge.

On day 103, the neonate was discharged in good clinical condition, with a surgical follow-up program.

### Follow-up assessments

At the 3-year follow-up assessment, the patient appeared in good clinical condition, with regular ponderal and linear growth and appropriate acquisition of developmental milestones. The abdominal wall had completely healed. Surgery was performed to repair bilateral inguinal hernias, with an uneventful postoperative course.

## Discussion

Although there has been significant progress in recent years in its management, omphalocele remains a complex condition that requires a multidisciplinary approach involving obstetricians, pediatric surgeons, neonatologists, and pediatricians.

Our report describes the therapeutic success of NPWT in a male newborn with a giant omphalocele, who underwent staged surgical treatment followed by NPWT for the management of a dehiscent surgical wound.

Congenital abdominal wall malformations require a prompt antenatal diagnosis (13), strict follow-up during pregnancy (including a fetal MRI, when appropriate), and multidisciplinary prenatal counseling for the parents (10).

In the literature, vaginal delivery is not contraindicated for small abdominal defects; however, a cesarean section is preferred in giant omphaloceles to reduce the risk of abdominal dystocia or other potential damage to the protruding organs (10, 14). A giant omphalocele is defined as a defect larger than 5 cm in diameter and/or containing as much as the 75% of the liver parenchyma (15). A liver-containing omphalocele places the infant at higher risk of pulmonary hypoplasia, pulmonary hypertension, and requiring mechanical ventilation; thus, it is mandatory to admit the baby to the neonatal intensive care unit after delivery (14, 16).

In this case, a giant omphalocele containing the liver and bowels was prenatally diagnosed at 13 weeks of gestational age. Therefore, after multidisciplinary counseling, an elective cesarean section was performed at a secondary center with both a neonatal intensive care unit and a pediatric surgery unit.

Since omphalocele is commonly associated with other disorders, our patient underwent a karyotype test for rearrangement of chromosomes 13, 18, and 21 and a test for microdeletions and microduplications related to Beckwith–Wideman and Silver–Russell syndromes, which were negative.

After neonatal stabilization, closure of the defect can be attempted through different, not fully standardized approaches.

Primary closure is still considered the first-choice treatment in clinically stable children with small abdominal wall defects and when the abdominal cavity is large enough to contain the herniated organs. In the case of giant omphaloceles, the treatment is not yet standardized, but the following staged closure technique is the standard approach in high-income countries: after construction of a silo bag, with or without application of a controlled traction on the abdominal wall, progressive reduction of the herniated organs into the abdominal cavity is gradually obtained while increasing abdominal capacity (17). In the final stage, 7–14 days after silo placement, direct closure of the abdominal wall layers can be performed, with or without the “component separation” method, rather than using a prosthetic patch to cover the wide muscle-fascial defect. Furthermore, skin flaps or tissue expanders have been used.

Non-operative delayed closure at a later age, by applying an initial topical antimicrobial dressing to induce epithelization of the hernial sac and followed by ventral hernia repair during infancy, could also be a valid alternative for larger defects, especially in middle- and low-income countries.

After surgery, in the case of complicated surgical wounds, the use of NPWT has been reported in the literature to facilitate wound healing using a specific approach (12). This approach removes the exudate, decreases the bacterial load, promotes the formation of granulation tissue and reperfusion, and reduces edema formation, ultimately leading to re-epithelialization of the abdominal wall. The use of NPWT appears to prevent wound dehiscence after primary surgery. Hospitalization periods ranging from 7 to 35 days after NPWT application have been reported (12).

Due to the large size of the defect, our patient was not a candidate for primary surgical repair. Thus, a staged closure was scheduled to avoid abdominal compartment syndrome and related unfavorable outcomes. A silo bag was initially fashioned, complete liver reduction was obtained, the defect was covered by a porcine dermal implant, and, finally, the abdominal wall was closed. Between the second and third interventions, NPWT was started and continued for 16 days. Thereafter, after fully closing the abdominal wall, NPWT was restarted due to partial dehiscence of the surgical wound and continued for 13 days. This approach was chosen based on expected beneficial effects, such as a reduction in exudate and edema, an increase in granulation tissue, and re-epithelialization.

The first reported use of NPWT in the treatment of three neonates with omphaloceles was published by Kilbride et al. (18). In 2018, Butler et al. published a report on the use of NPWT in the management of a neonate with gastroschisis after silo bag positioning and secondary surgery failure (19). In 2021, Tri et al. reported the use of NPWT in three different cases of a neonate with an omphalocele, with a mean hospitalization period of 71 days (12).

Continuous negative pressure suction therapy for open wounds has been found to be effective in inducing granulation tissue formation, limiting the spread of infection and liquid loss, and reducing wound healing time. The use of NPWT in omphaloceles is analogous to open wound management, with similar results. The expansion of granulation tissue seems to play a crucial role in protecting the underlying viscera and limiting infections. Moreover, NPWT appears to reduce organ edema and increase abdominal compliance, allowing the organs to be reduced into the abdomen (12, 20, 21). In our case, NPWT was performed in two distinct stages, for a cumulative duration of 29 days, and led to optimal clinical results.

## Conclusions

In conclusion, this report highlights the importance of a detailed antenatal diagnosis and performing a prompt neonatal assessment in patients with omphaloceles, as these lead to timely diagnostic and therapeutic approaches. Furthermore, this case underlines the efficacy of NPWT in the management of an abdominal surgical wound after omphalocele repair, given the reduction in dressing frequency, healing time, and related stress.

## Data Availability

The original contributions presented in the study are included in the article/Supplementary Material, further inquiries can be directed to the corresponding author.
